# Anesthetic management of a patient with Bardet-Biedl syndrome undergoing renal transplantation: A case report

**DOI:** 10.1097/MD.0000000000022300

**Published:** 2020-09-18

**Authors:** Ferda Yaman, Nedim Çekmen

**Affiliations:** a University of Eskişehir Osmangazi, Department of Anesthesiology and Reanimation, Faculty of Medicine, Eskişehir; b Department of Anesthesiology and Reanimation, Istanbul Nisantasi University, İstanbul, Turkey.

**Keywords:** Bardet-Biedl syndrome, general anesthesia, renal transplantation

## Abstract

**Introduction:**

Bardet-Biedl syndrome, which compromises airway management and the cardiovascular and renal systems, is a rare ciliopathic syndrome characterized by multisystem involvement and varying genetic etiologies and clinical manifestations.

**Patient concerns:**

A 13-year-old female patient had a history of chronic renal failure, hypothyroidism, mental retardation, hypogonadotropic hypogonadism, obesity, and retinitis pigmentosa and was undergoing 4-hour hemodialysis 3 days a week.

**Diagnosis:**

We diagnosed Bardet-Biedl syndrome based on the results of genetic tests.

**Interventions:**

We performed renal transplantation under general anesthesia while considering the perioperative risks of airway obstruction and hypothermia.

**Outcomes:**

Multidisciplinary preoperative evaluation is crucial to avoid perioperative complications. The risk of an obstructed airway should be considered. Hypothyroidism is a rare consequence of Bardet-Biedl syndrome. Rocuronium and sugammadex are safe for anesthetic management during renal transplantation to address Bardet-Biedl syndrome.

**Conclusion:**

Safe anesthetic management can be achieved with the rigorous preoperative assessment of perioperative complications.

## 1 Introduction

Also known as Laurance-Moon-Bardet-Biedl syndrome, Bardet-Biedl syndrome (BBS) is a rare autosomal recessive disorder with a prevalence of 1:160,000 people in Northern European populations and 1:13,500 in the Arab population. The syndrome affects multiple organ systems and induces obesity, mental retardation, retinitis pigmentosa, polydactyly, hypogonadism, and renal abnormalities.^[1]^ Patients with BBS have to undergo multiple operations during their lives to prolong their life expectancy.^[2]^ In addition to its primary diagnostic criteria, BSS involves secondary characteristics such as left ventricular hypertrophy, hepatic fibrosis, high palate, diabetes mellitus, nephrogenic diabetes insipidus, renal failure, cataract, speech disorder, and developmental delay.^[3]^ The literature features a number of studies on anesthetic management during the treatment of BBS. The present case report presents our perioperative anesthetic approach during a renal transplantation for a patient diagnosed with BBS and considers the patient's clinical course in the context of the literature.

## 2 Case report

This case report was submitted for publication after the family was informed of our intentions, and their permission was obtained and also written informed consent was obtained from the patient for publication of the case details. During follow-up of the patient's hypothyroidism, we found that the 13-year-old female patient had a creatinine level of 7.42 mg/dl and urea concentration of 172 mg/dl. Renal ultrasonography revealed abnormally small bilateral kidney sizes; considered in the context of concomitant diseases like obesity, hypogonadotropic hypogonadism, retinitis pigmentosa, and mild mental retardation, this finding informed a preliminary diagnosis of Bardet-Biedl syndrome, which was confirmed with genetic tests. The patient had undergone hemodialysis for 4 hours, 3 days of a week since 10 years old. She was approved by the commission of transplantation to receive kidney transplantation. The patient was prescribed levothyroxine 50 mg/day for hypothyroidism.

The patient had poor speech and school performance and a history of frequent urinary tract infections. Physical examination revealed a Mallampati Classification of III, submental fullness, short neck, large tongue, a thyromental distance of 4.5 cm, a sternomental distance of 5.5 cm, a trachea positioned along the midline, the non-palpable thyroid gland, and the absence of chest deformity. Preoperative laboratory tests yielded the following values: creatinine: 4.59 mg/dl, urea: 92 mg/dl, TSH: 5.88 mIU/L, and free T3 and T4 levels within normal limits. The echocardiographic examination revealed a minimal insufficiency in the tricuspid valve and normal ventricular functions.

Before anesthesia was administered, devices were prepared in anticipation of airway obstruction. After anesthesia was induced with the intravenous administration of 1 mg/kg propofol and 1 mg/kg rocuronium, the Cormack Lehane Score was evaluated as Grade 2b (only the posterior end of the glottis was visible) with direct laryngoscopy, and intubation was performed on the first attempt. The anesthesia was maintained with the intravenous administration of propofol and remifentanil without any hemodynamic complications. The operation was completed in 180 minutes. Heat monitorization was performed for the patient: normothermia was maintained with a heater blanket during the surgery, and precautions were taken against hypothermia such as warmed intravenous infusion. Decurarization was ensured with sugammadex. The patient was extubated without any problems and was transferred to the transplantation unit. Postoperative acute pain control was maintained with 15 mg/kg paracetamol and 1 mg/kg tramadol. Immunosuppression was achieved with tacrolimus, and the patient was discharged after 1 week.

## 3 Discussion

BBS is an autosomal recessive genetic disorder with multisystemic involvement. The condition is characteristic of a group of disorders referred to as “ciliopathies”^[4]^: the result of mutations of more than 20 different genes that play important roles in the structure of cilia.^[5]^ In clinical practice, ciliopathies are characterized by an immotile cilia defect, retinitis pigmentosa, polydactyly, situs inversus, learning disability, cystic kidney, and liver and pancreas cysts. A total of 90% of patients with BBS exhibited abnormally small kidneys, irregular parenchymal loss, distortion of the calyx, and urological abnormalities that included medullary cysts. BBS reportedly causes renal tubular dysfunction, decreased urinary concentration, glucosuria, and aminoaciduria in 50% of afflicted patients. Renal insufficiency is the most contributory cause of early mortality and morbidity resulting from BBS: 25% of patients with BBS die around the age of 44.^[6]^ Renal transplantation offers a better quality of life for such patients.

In patients with BBS, the risk of airway obstruction is increased because of facial dysmorphism, high palate, morbid obesity, and dental anomalies. Intubation is easier for pediatric patients under the age of 18 because progressive facial anomalies and morbid obesity compromise intubation in older patients.^[7]^ Our patient was diagnosed with BBS at the age of 10. While she had central obesity, her body mass index was within normal limits. The patient had a Mallampati score 3 in the preoperative period, and advanced airway devices such as a video laryngoscope and fiberoptic laryngoscopy were prepared for in anticipation of airway obstruction. Intubation was performed with direct laryngoscopy on the first attempt, and the patient was easily ventilated with double hand mask ventilation. A total of 67% of patients with BBS above the age of 18 years are intubated with indirect laryngoscopy techniques, 40% with the awake fiberoptic intubation technique, and 27% with video laryngoscope.^[7]^

BBS patients mat present with comorbid hypertension (50%) and diabetes mellitus (32%).^[8]^ Subclinical hypothyroidism is detected in 24 of 125 patients (19.4%).^[9]^ The blood sugar and blood pressure measurements of our patient were normal, as she had no diabetes mellitus or hypertension. The patient's hypothyroidism was addressed with 50 mg levothyroxine, which restored normal levels of free T3 and T4.

Cardiac involvement may occur in patients with BBS and concomitant congenital heart disease, dilated cardiomyopathy, atrial septal defects, ventricular septal defects, large vessel transposition, patent ductus arteriosus, dextrocardia, or hypoplastic aorta; these comorbidities are most frequently presented by male patients.^[7]^ The literature features reports of patients with BBS and left ventricular hypertrophy, biventricular hypertrophy, and pulmonary hypertension.^[8]^ Such patients must undergo echocardiography in the preoperative period. In the case of our patient, the echocardiographic examination revealed minimal tricuspid regurgitation.

Since patients with BBS also suffer from visual difficulties, mental retardation, and behavioral disorders, different levels of premedication are required. As the present patient had central obesity and a short neck, 0.05 mg/kg midazolam was preoperatively administered intravenously at the surgical area. Her family accompanied her to the entrance of the surgical room to prevent the onset of agitation.

Since the patient was scheduled to undergo renal transplantation, we chose to administer short-acting anesthetic drugs. Total intravenous anesthesia was selected to maintain anesthesia because of its pharmacokinetic properties. Remifentanil has an ester structure and is metabolized by ester hydrolysis; hence, it is characterized by extrarenal excretion and short duration of action. Propofol prevents postoperative nausea and vomiting and is an appropriate intravenous anesthetic for uremic patients.^[10]^

Rocuronium was preferred as a muscle relaxant because of its rapid onset of action and its specific antagonist. It can be used to treat patients with severe renal failure and avoid respiratory complications. However, its cholinergic side effects including, bradycardia, salivation, bronchoconstriction, nausea, vomiting, and hypotension, may cause undesirable effects in patients with ciliary dyskinesia. Further, the metabolism and redistribution of cholinesterase inhibitors are delayed in cases of severe renal failure, which extends the duration of their effects and increases the risk of the attendant side effects.

The use of sugammadex in patients with renal transplantation or renal insufficiency remains controversial. Sugammadex is renally excreted; up to 97% is cleared within 24 hours in cases of normal renal function. In cases of severe renal failure; however, the clearance of sugammadex is reduced by 17-fold, and the elimination half-life is increased 15-fold and not licensed for severe renal failure.^[11,12]^ Ciliary dysfunction may cause difficulties in removing secretions following general anesthesia. Hence, avoiding the residual block becomes even more important for ensuring the safety of the patient's airway. Considering the profit–loss rate, sugammadex was a good option for performing renal transplantation in our patient with ciliary dysplasia.

In conclusion, safe anesthetic management can be achieved when treating BBS by preoperatively anticipating and planning for perioperative complications, examining the airway, and carefully considering cardiovascular abnormalities, renal function, and endocrine function.

## Author contributions

FY collected and recorded the original data, analyzed the case and was a major contributor in writing the manuscript, NÇ guided and revised all the work.

## Abbreviations

Abbreviation: BBS = Bardet-Biedl Syndrome.
